# Out-of-Pocket Costs of Diagnostic Breast Imaging Services After Screening Mammography Among Commercially Insured Women From 2010 to 2017

**DOI:** 10.1001/jamanetworkopen.2021.21347

**Published:** 2021-08-17

**Authors:** Kathryn P. Lowry, Sarah Bell, A. Mark Fendrick, Ruth C. Carlos

**Affiliations:** 1 Department of Radiology, University of Washington, Seattle Cancer Care Alliance, Seattle; 2 Program for Women’s Health Effectiveness Research, University of Michigan, Ann Arbor; 3 Department of Obstetrics and Gynecology, University of Michigan, Ann Arbor; 4 Department of Medicine, University of Michigan, Ann Arbor; 5 Department of Radiology, University of Michigan, Ann Arbor

## Abstract

This cross-sectional study evaluates the out-of-pocket costs of diagnostic breast imaging services incurred by commercially insured women who underwent additional imaging evaluation and procedures after screening mammography.

## Introduction

The Affordable Care Act (ACA) eliminated out-of-pocket costs (OOPCs) for nearly all women who undergo screening mammography.^1^ However, this mandate does not prohibit OOPCs for additional breast imaging examinations or procedures, which occur after greater than 10% of screening examinations.^2^ Because screening examinations may result in unanticipated financial consequences for patients, we examined trends in OOPCs for commercially insured women who underwent additional breast imaging evaluations or procedures after screening mammography.

## Methods

This cross-sectional study was deemed exempt from University of Michigan Institutional Review Board approval owing to use of deidentified data and followed the Strengthening the Reporting of Observational Studies in Epidemiology (STROBE) reporting guideline. We performed a retrospective analysis using a national commercial claims database (OptumInsight, Eden Prairie, Minnesota) with individual-level demographic information and inpatient, outpatient, and pharmacy claims for health care plan members residing in all 50 US states. Claims information included both OOPCs for plan members (eg, deductibles, copayments, and coinsurance) and total standardized reimbursements.

We included women ages 40 to 64 years enrolled in employer-based health care plans between 2010 and 2017 who underwent screening mammography and additional breast imaging examinations or procedures within 11 months of the screening mammogram. Exclusion criteria were fewer than 11 months of continuous enrollment in a single plan, 2 screening mammograms within 11 months, or prior breast cancer or mastectomy (eFigure in the Supplement).

Total and OOPCs were quantified and stratified by type of imaging received after screening mammography, including diagnostic mammography (DM), ultrasonography (US), magnetic resonance imaging (MRI), and biopsy (eTable in the Supplement), expressed in 2018 US dollars. Annual median cost-sharing per woman was calculated. Analyses were conducted using SAS, version 9.4 (SAS Institute Inc).

## Results

Of 6 216 270 screening mammography examinations, 993 005 (16%) were followed by additional breast imaging examinations or procedures. After applying exclusions, the final cohort included 325 900 women with 418 378 additional breast imaging examinations or procedures.

Out-of-pocket costs varied substantially across women and type of imaging received and generally increased over time (Table). For example, the median OOPC for SM+DM+US was $0 (interquartile range [IQR], $0-$72.26) in 2010 and increased to $23.44 (IQR, $0-$144.91) in 2017. The median OOPCs for all pathways with MRI increased from $24.49 (IQR, $0-$171.43) in 2010 to $47.50 (IQR, $0-$253.54) in 2017. Women who underwent biopsy had higher OOPCs than those who did not: median OOPCs for SM+DM+US+biopsy increased from $94.60 (IQR, $0-$290.63) in 2010 to $170.80 (IQR, $38.62-$475.29) in 2017.

**Table.** Out-of-Pocket Costs for Breast Imaging Examinations and Procedures During the 11-Month Period After Screening Mammography

Imaging type	Out-of-pocket costs, median (IQR), $^a^							
	2010 (n = 18 757)	2011 (n = 29 333)	2012 (n = 35 271)	2013 (n = 43 287)	2014 (n = 51 025)	2015 (n = 77 525)	2016 (n = 77 508)	2017 (n = 85 672)
SM+DM	0 (0-45.49)	0 (0-48.36)	0 (0-50.21)	0 (0-54.82)	0 (0-59.57)	0 (0-63.14)	0 (0-66.17)	0 (0-73.37)
SM+DM+US	0 (0-72.26)	0 (0-71.64)	0 (0-81.21)	0 (0-88.75)	0 (0-102.36)	10.81 (0-128.75)	10.45 (0-134.35)	23.44 (0-144.91)
SM+US	0 (0-37.49)	0 (0-26.05)	0 (0-30.70)	0 (0-38.55)	0 (0-44.24)	0 (0-74.90)	0 (0-76.28)	0 (0-69.16)
SM+MRI^b^	24.49 (0-171.43)	30.56 (0-235.94)	27.36 (0-231.40)	28.66 (0-183.30)	33.77 (0-237.82)	43.49 (0-266.17)	43.62 (0-257.20)	47.50 (0-253.54)
SM+DM+biopsy	130.40 (15.30-361.97)	122.52 (0-372.27)	118.82 (0-384.05)	98.09 (3.40-329.73)	113.19 (12.30-383.32)	117.07 (17.82-393.32)	129.99 (14.53-393.33)	165.22 (35.15-454.74)
SM+DM+US+biopsy	94.60 (0-290.63)	89.89 (0-322.71)	92.73 (11.33-316.18)	96.62 (7.15-357.99)	129.94 (22.17-416.21)	152.48 (28.45-455.32)	146.52 (27.09-450.92)	170.8 (38.62-475.29)
SM+US+biopsy	41.79 (0-177.30)	44.42 (0-166.36)	46.28 (0-194.50)	44.94 (0-205.62)	38.9 (0-210.69)	69.89 (0-264.79)	52.43 (0-247.00)	50.98 (0-236.01)
SM+MRI+biopsy^b^	88.68 (15.88-349.52)	85.93 (0-401.14)	104.62 (0-400.13)	162.23 (43.27-536.90)	203.05 (36.03-609.85)	163.94 (32.46-630.12)	162.21 (30.33-588.13)	190.03 (17.70-581.00)
Any imaging without biopsy	0 (0-53.49)	0 (0-53.60)	0 (0-57.60)	0 (0-62.51)	0 (0-70.18)	0 (0-91.34)	0 (0-96.77)	7.05 (0-100.70)
Any imaging with biopsy	91.14 (0-301.73)	85.68 (0-314.01)	92.51 (0-312.64)	89.43 (0-325.08)	110.85 (5.45-373.10)	129.84 (18.25-415.47)	129.82 (13.47-410.67)	151.95 (25.49-436.79)
All pathways	0 (0-74.54)	0 (0-74.59)	0 (0-77.43)	0 (0-81.08)	0 (0-89.57)	11.68 (0-124.43)	12.69 (0-128.65)	18.13 (0-132.39)

Abbreviations: DM, diagnostic mammography; IQR, interquartile range; MRI, magnetic resonance imaging; SM, screening mammography; US, ultrasonography.

^a^ All out-of-pocket costs were highly right skewed, with skewedness values ranging from 1.9 to 10.9 across imaging types.

^b^ All pathways with MRI performed, including DM and/or US.

Cost sharing also increased throughout the study period (Figure). Among women receiving SM+DM+US, the median percentage of costs shared increased from 0% (IQR, 0%-20.5%) in 2010 to 7.5% (IQR, 0%-47.3%) in 2017. Shared costs nearly doubled for women who received a biopsy; for example, for women receiving SM+DM+US+biopsy, the median percentage of costs shared increased from 9.6% (IQR, 0%-30.2%) to 18.2% (IQR, 3.7%-46.2%) of the total costs.

**Figure.**
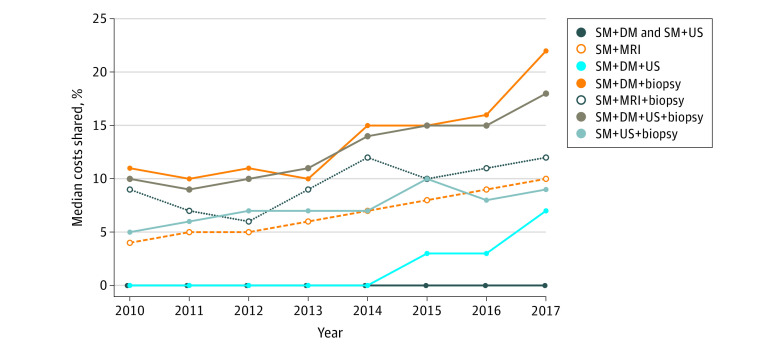
Median Cost Sharing for Breast Imaging Examinations and Procedures Performed Within 11 Months of Screening Mammography by Year, Stratified by Imaging Type Cost sharing was defined as the percentage of total costs that were out-of-pocket for the health care plan member, including deductibles, coinsurance, and copayments. DM indicates diagnostic mammography; MRI, magnetic resonance imaging; SM, screening mammography; US, ultrasonography.

## Discussion

Although the ACA largely eliminated OOPCs for screening mammography, our findings suggest that among commercially insured women ages 40 to 64 years, OOPCs for additional breast imaging evaluations and procedures after screening are common, nontrivial, and increasing. This trend coincides with the rapid rise in high-deductible health care plans that has been observed during the same time frame as the study period.^3^

This study has some limitations. We did not include OOPCs for related care such as office visits and pathology expenses, likely underestimating the total patient contributions. In addition, we could not distinguish between diagnostic evaluations for abnormal screening vs those for symptoms. We were also unable to discern the number of women who had an abnormal screening but did not undergo a subsequent evaluation or procedure.

Consumer cost sharing is associated with decreased use of evidence-based medical care.^4^ Although the association between OOPCs and receipt of one-time diagnostic testing has not been described (to our knowledge), it is possible that higher cost sharing could deter women from undergoing diagnostic evaluation following screening mammography, thus undermining the goal of the ACA to remove barriers to screening. The benefit design of health care plans must acknowledge that cancer screening often requires multiple steps and remove financial barriers for patients to complete the screening process.

## Notes

Supplement. **eFigure.** Flow Diagram of Screening Episode Selection With Exclusions **eTable.** Diagnosis and Procedural Codes Used to Identify Claims for Breast Imaging Examinations and Procedures

## References

[1] Carlos RC, Fendrick AM, Kolenic G, . Breast screening utilization and cost sharing among employed insured women after the Affordable Care Act. J Am Coll Radiol. 2019;16(6):788-796. doi:10.1016/j.jacr.2019.01.028 30833168 PMC7384237

[2] Lehman CD, Arao RF, Sprague BL, . National performance benchmarks for modern screening digital mammography: update from the Breast Cancer Surveillance Consortium. Radiology. 2017;283(1):49-58. doi:10.1148/radiol.2016161174 27918707 PMC5375631

[3] Cohen RA, Martinez ME, Zammitti EP. Health insurance coverage: early release of estimates from the National Health Interview Survey, January–March 2018. National Center for Health Statistics. Accessed May 26, 2021. https://www.cdc.gov/nchs/data/nhis/earlyrelease/Insur201808.pdf

[4] Neugut AI, Subar M, Wilde ET, . Association between prescription co-payment amount and compliance with adjuvant hormonal therapy in women with early-stage breast cancer. J Clin Oncol. 2011;29(18):2534-2542. doi:10.1200/JCO.2010.33.3179 21606426 PMC3138633

