# Supplementary material for: Out-of-Pocket Costs of Diagnostic Breast Imaging Services After Screening Mammography Among Commercially Insured Women From 2010 to 2017
Source: JAMA Netw Open. 2021 Aug 17;4(8):e2121347. doi: 10.1001/jamanetworkopen.2021.21347 (PMC8371567; doi:10.1001/jamanetworkopen.2021.21347)
Supplement: Supplement. — eFigure. Flow Diagram of Screening Episode Selection With Exclusions eTable. Diagnosis and Procedural Codes Used to Identify Claims for Breast Imaging Examinations and Procedures [file jamanetwopen-e2121347-s001.pdf]

Supplementary Online Content

Lowry KP, Bell S, Fendrick AM, Carlos RC. Out-of-pocket costs of diagnostic breast imaging services after screening mammography among commercially insured women from 2010 to 2017. *JAMA Netw Open*. 2021;4(8):e2121347. doi:10.1001/jamanetworkopen.2021.21347

eFigure. Flow Diagram of Screening Episode Selection With Exclusions

eTable. Diagnosis and Procedural Codes Used to Identify Claims for Breast Imaging Examinations and Procedures

This supplementary material has been provided by the authors to give readers additional information about their work.

© 2021 Lowry KP et al. *JAMA Network Open*.

eFigure. Flow Diagram of Screening Episode Selection With Exclusions

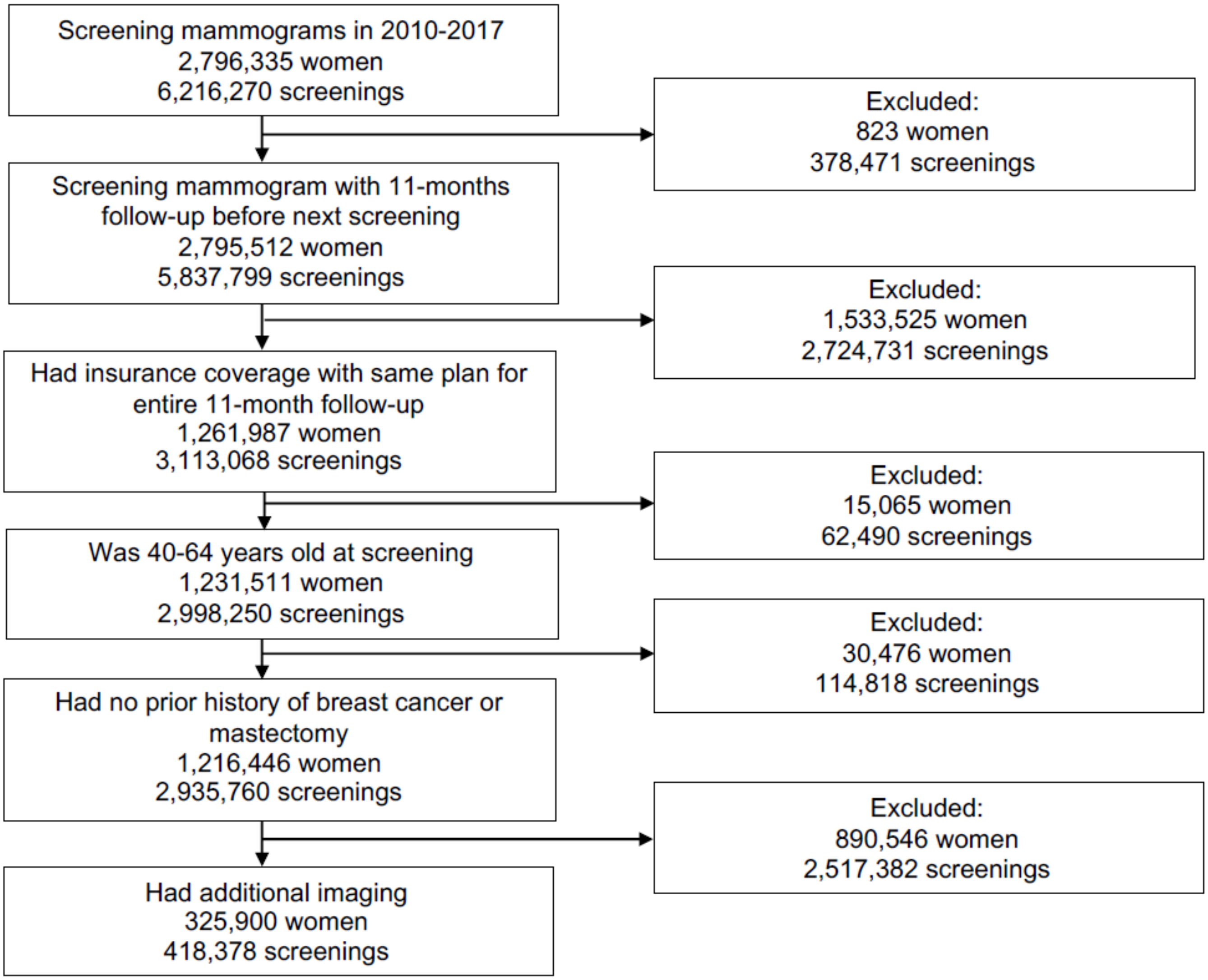

© 2021 Lowry KP et al. *JAMA Network Open*.

eTable. Diagnosis and Procedural Codes Used to Identify Claims for Breast Imaging Examinations and Procedures

Diagnosis/Procedure	ICD-9 and ICD-10 Codes	CPT/HCPCS Codes
Mastectomy	8541, 8542, 8543, 8544, 8545, 8546, 8547, 8548, 0HTT0ZZ, 0HTU0ZZ, 0HTV0ZZ, 07T50ZZ, 07T60ZZ, 07T70ZZ, 07T80ZZ, 07T90ZZ, 0H0T0JZ, 0H0T3JZ, 0H0U0JZ, 0H0U3JZ, 0H0V0JZ, 0H0V3JZ, 0HBT0ZZ, 0HBT3ZZ, 0HBU0ZZ, 0HBU3ZZ, 0HBV0ZZ, 0HBV3ZZ, 0HRT0JZ, 0HRT3JZ, 0HRU0JZ, 0HRU3JZ, 0HRV0JZ, 0HRV3JZ, 0KTH0ZZ, 0KTJ0ZZ, Z901, Z9010, Z9011, Z9012, Z9013	19160, 19162, 19180, 19182, 19200, 19220, 19240, 19301, 19302, 19303, 19304, 19305, 19306, 19307
Screening mammogram		G0202, 77057, 76092, 76083, 77067
Diagnostic mammogram		76082, 76090, 76091, 77055, 77056, G0204, G0206, 77065, 77066
Magnetic resonance imaging		0159T, 77058, 77059, 77046, 77047, 77048, 77049
Biopsy		10021, 19100, 19102, 19103, 19290, 19291, 19295, 76095, 76096, 76098, 76942, 77031, 77032, 19081, 19082, 19083, 19084, 19085, 19086, 10005, 10006
Ultrasound		76645, 76641, 76642
History of breast cancer	1740, 1741, 1742, 1743, 1744, 1745, 1746, 1748, 1749, V103, 2330, Z853, D0590, D0591, D0592	

Abbreviations: CPT, Current Procedural Terminology; HCPC, Healthcare Common Procedural Coding;ICD, International Classification of Diseases.

© 2021 Lowry KP et al. *JAMA Network Open*.
